# Beneficial effects of increased lysozyme levels in Alzheimer's disease modelled in *Drosophila melanogaster*

**DOI:** 10.1111/febs.13830

**Published:** 2016-10-06

**Authors:** Linnea Sandin, Liza Bergkvist, Sangeeta Nath, Claudia Kielkopf, Camilla Janefjord, Linda Helmfors, Henrik Zetterberg, Kaj Blennow, Hongyun Li, Camilla Nilsberth, Brett Garner, Ann‐Christin Brorsson, Katarina Kågedal

**Affiliations:** ^1^ Division of Cell BiologyDepartment of Clinical and Experimental MedicineFaculty of Medicine and Health SciencesLinköping UniversitySweden; ^2^ Division of Molecular BiotechnologyDepartment of Physics, Chemistry and BiologyLinköping UniversitySweden; ^3^ Clinical Neurochemistry LaboratoryDepartment of Neuroscience and PhysiologySahlgrenska University HospitalMölndalSweden; ^4^ UCL Institute of NeurologyLondonUK; ^5^ Illawarra Health and Medical Research InstituteUniversity of WollongongAustralia; ^6^ Department of Acute Internal Medicine and Geriatrics and Department of Clinical and Experimental MedicineLinköping UniversitySweden; ^7^ School of Biological SciencesUniversity of WollongongAustralia

**Keywords:** Alzheimer's disease, amyloid‐β, *Drosophila*, lysozyme

## Abstract

Genetic polymorphisms of immune genes that associate with higher risk to develop Alzheimer's disease (AD) have led to an increased research interest on the involvement of the immune system in AD pathogenesis. A link between amyloid pathology and immune gene expression was suggested in a genome‐wide gene expression study of transgenic amyloid mouse models. In this study, the gene expression of lysozyme, a major player in the innate immune system, was found to be increased in a comparable pattern as the amyloid pathology developed in transgenic mouse models of AD. A similar pattern was seen at protein levels of lysozyme in human AD brain and CSF, but this lysozyme pattern was not seen in a tau transgenic mouse model. Lysozyme was demonstrated to be beneficial for different *Drosophila melanogaster* models of AD. In flies that expressed Aβ_1‐42_ or AβPP together with BACE1 in the eyes, the rough eye phenotype indicative of toxicity was completely rescued by coexpression of lysozyme. In *Drosophila* flies bearing the Aβ_1‐42_ variant with the Arctic gene mutation, lysozyme increased the fly survival and decreased locomotor dysfunction dose dependently. An interaction between lysozyme and Aβ_1‐42_ in the *Drosophila* eye was discovered. We propose that the increased levels of lysozyme, seen in mouse models of AD and in human AD cases, were triggered by Aβ_1‐42_ and caused a beneficial effect by binding of lysozyme to toxic species of Aβ_1‐42_, which prevented these from exerting their toxic effects. These results emphasize the possibility of lysozyme as biomarker and therapeutic target for AD.

**Abbreviations**
AD: Alzheimer's disease
Aβ: amyloid‐β
AβPP: amyloid‐β precursor protein
BACE1: β‐site AβPP‐cleaving enzyme 1
CSF: cerebrospinal fluid
PSEN1: presenilin 1
WT: wild‐type

## Introduction

Two of the main pathological hallmarks of Alzheimer's disease (AD) are extracellular accumulation of amyloid plaques, which consists of amyloid‐β (Aβ) peptides, and intracellular neurofibrillary tangles composed of hyperphosphorylated tau 1. The amyloid plaques were previously considered the foremost neurotoxic agents in AD, but increasing evidence suggests that small diffusible Aβ aggregates (referred to as oligomers) are the principal cytotoxic species as these correlate better with synaptic loss and cognitive impairment than the plaques 2. Aβ peptides are produced from sequential cleavage of the amyloid‐β precursor protein (AβPP) by β‐secretase activity of the β‐site AβPP‐cleaving enzyme 1 (BACE1) and the γ‐secreatase complex, where presenilin 1 (PSEN1) is the catalytic subunit 3. Although Aβ_1‐40_ is the predominant secreted form, the longer Aβ_1‐42_ has a more prominent part in AD as it is more aggregation prone and forms toxic oligomers more easily 4. Besides Aβ and tau, it is well established that neuroinflammation is involved in AD. Activated astrocytes and microglia cells surround Aβ plaques together with various inflammatory mediators 5, 6. In addition, genetic studies show upregulation of several genes involved in inflammation, especially complement activation and prostaglandin synthesis, during incipient AD 7 and there is data which demonstrate inflammatory processes before tangles and neurodegeneration are apparent 8. However, microglial activation also demonstrates a protective function of a triggered immune response in AD, as microglial activation mediates phagocytosis and clearance of Aβ 9.

Lysozyme, which belongs to the innate immune system, is upregulated in cerebrospinal fluid (CSF) from AD patients and inhibits the formation of toxic Aβ oligomers 10, 11, 12. Lysozyme overexpression in Aβ transgenic *Drosophila melanogaster* rescues both the survival and the activity of the Aβ flies 10. Lysozyme is a glucoside hydrolase able to hydrolyse peptidoglycans found in the cell walls of bacteria 13. It is secreted from macrophages and microglia, and it is abundant in various secretions such as tears, saliva, milk and CSF 14. The aim of this study was to further investigate the implication of lysozyme in AD. Lysozyme gene expression was investigated using a database of a genome‐wide gene expression study of wild‐type (WT) and five mouse models of AD (mutant human AβPP, mutant human PSEN1, homozygous and heterozygous expressed AβPP–PSEN1 and mutant human TAU) 15, and a database of AD patient brain tissue 16. The levels of lysozyme protein were investigated in brain tissue from transgenic AD mice and AD patients. An increased lysozyme expression was found both at mRNA and protein level in AD brain tissue of both mice and humans. In order to investigate the impact of lysozyme expression during AD, three different *Drosophila* models of AD were used. Beneficial effects of lysozyme in these different *Drosophila* models were discovered; in flies that expressed Aβ_1‐42_ individually or AβPP together with BACE1 (AβPP–BACE1) in the fly eyes, the AD phenotype was completely rescued by lysozyme. In flies carrying the highly toxic Aβ peptide with the Arctic mutation (E22G), lysozyme increased the fly survival and improved the locomotor behaviour in a dose‐dependent manner. These results imply that lysozyme has a protective effect on Aβ toxicity and could function as a new therapeutic strategy for AD.

## Results

### Lysozyme is increased in brains of transgenic AD mice

**Figure 1:**
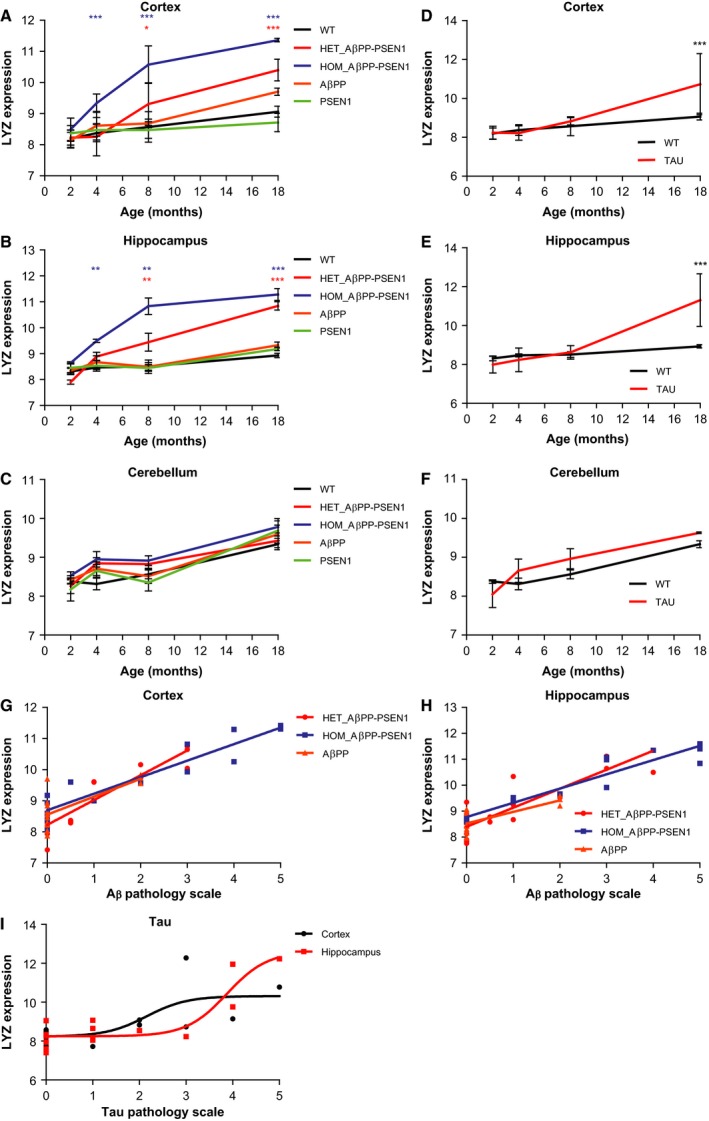
Lysozyme mRNA expression is increased in brains of transgenic AD mice. (A–C) Lysozyme mRNA expression (LYZ) in the cortex, hippocampus and cerebellum of wild‐type (WT) mice and amyloid transgenic mice expressing human AβPP or human PSEN1 or both genes homo‐ or heterozygously (HOM_AβPP–PSEN1 and HET_AβPP–PSEN1) at 2, 4, 8 and 18 months of age. Significant increases of lysozyme in HOM_AβPP–PSEN1 and HET_AβPP–PSEN1 compared with WT mice are denoted with asterix (*). Significant differences were determined by two‐way ANOVA with Tukey's *post hoc* test, *P ≤ 0.05, **P ≤ 0.01, ***P ≤ 0.001. Mean and SD are presented at the different ages, *n* = 4. (D–F) Lysozyme mRNA expression in the cortex, hippocampus and cerebellum in tau transgenic mice at 2, 4, 8 and 18 months of age. Significant increases of lysozyme in TAU mice compared with WT mice are denoted with asterix (*). Significant differences were determined by two‐way ANOVA with Tukey's *post hoc* test. Mean and SD are presented at the different ages, *n* = 4. (G,H) Correlation analysis of lysozyme mRNA expression and Aβ pathology in the cortex and hippocampus of homozygous or heterozygous AβPP–PSEN1 mice and AβPP mice using the Pearson correlation coefficient. (I) Correlation analysis of lysozyme mRNA expression and tau pathology in cortex and hippocampus of tau transgenic mice using nonlinear regression.

To investigate whether the mRNA expression of lysozyme is changed during AD progression, we used data from the publicly available database www.mouseac.com on five different amyloid or tau mouse dementia models. The mouse models were analysed at the ages 2, 4, 8 and 18 months 15. Homozygous and heterozygous expression of human AβPP, with the Swedish mutation in combination with mutant human PSEN1 (AβPP–PSEN1), leads to plaque formation at 4 and 8 months, respectively, mutant AβPP expressed separately leads to plaques first at 18 months and mutant PSEN1 expressed separately has no plaque pathology. The mutant human heterozygous TAU mice demonstrate tangles at 8 months. The gene expression of lysozyme in the homozygous AβPP–PSEN1 mice was found to be significantly increased at 4 months in cortex (Fig. 1A) and hippocampus (Fig. 1B) and in heterozygous AβPP–PSEN1 mice at 8 months compared to WT mice (Fig. 1A,B). Lysozyme levels were unchanged in cerebellum of both homozygous and heterozygous AβPP–PSEN1 mice (Fig. 1C). In AβPP mice, there was a trend of increased lysozyme gene expression in cortex at 18 months, but not in hippocampus and no change was detected in PSEN1 mice (Fig. 1A–C). We next investigated the correlation between lysozyme gene expression and Aβ pathology in the cortex (Fig. 1G) and hippocampus (Fig. 1H) of these mice. Both heterozygous and homozygous AβPP–PSEN1 mice showed a strong and significant linear correlation in the cortex (*r* = 0.91 and 0.94 respectively) and in hippocampus (*r* = 0.86 and 0.95 respectively) (Fig. 1G,H). Mice only expressing AβPP exhibited a strong and significant correlation as well, both in cortex and hippocampus, albeit weaker than for the double transgenic mice (*r* = 0.74 and 0.77 respectively) (Fig. 1G,H). Tau transgenic mice showed an increase in lysozyme expression in cortex (Fig. 1D) and hippocampus (Fig. 1E) only after 18 months. In the cerebellum of the tau transgenic mice, no change in lysozyme gene expression was detected (Fig. 1F). A sigmoid correlation relationship between lysozyme levels and tau pathology was demonstrated in hippocampus (Fig. 1I; *R* ^2^ = 0.81), while this sigmoid correlation relationship was weaker in cortex (Fig. 1I; *R* ^2^ = 0.52). These results demonstrate that the increase of Aβ plaque pathology can trigger lysozyme expression in the AD mice brain, while tau pathology has no immediate impact on lysozyme expression.

**Figure 2:**
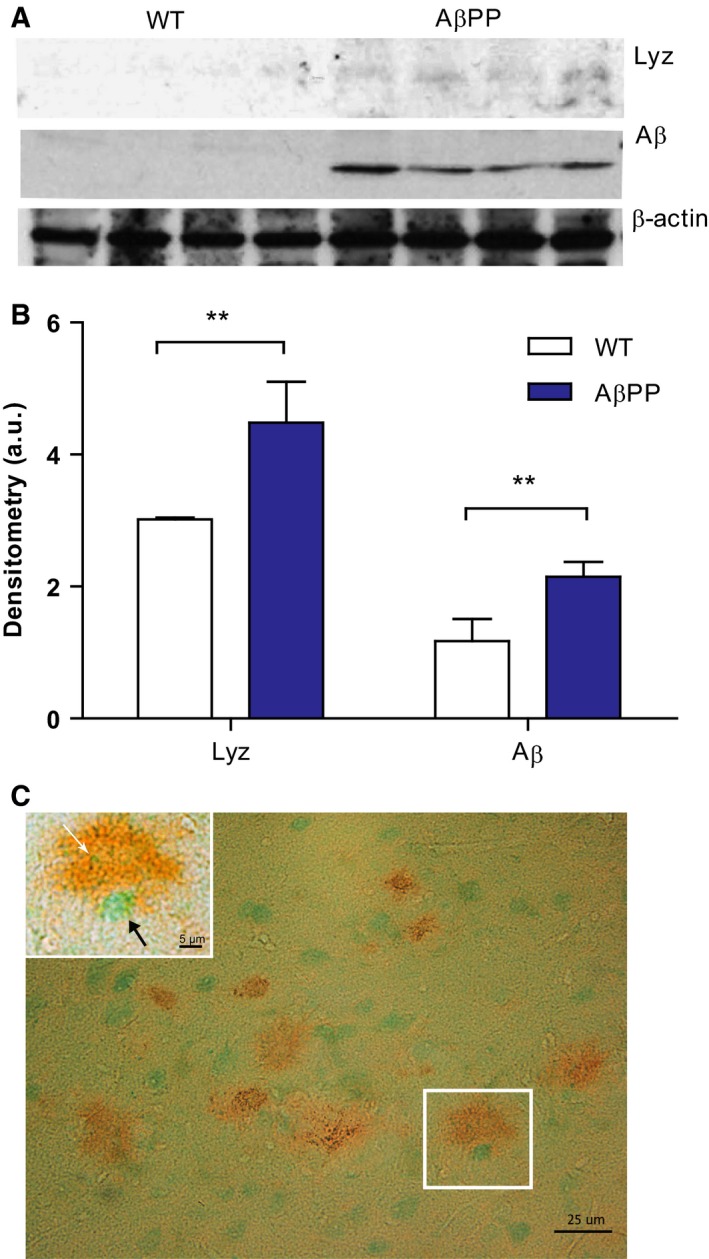
Lysozyme protein expression is increased in brains of transgenic AD mice. (A) Representative western blot of brain homogenates from 12‐month‐old WT and AβPP transgenic mice analysed for lysozyme, Aβ and β‐actin. (B) Densitometric quantification of the western blot. Significant differences were determined by Student's *t*‐test. Error bars represent mean ± SD,* n* = 4. (C) Immunohistochemistry of brain tissue prepared from 15‐month‐old transgenic AβPP_S__we_ mice, stained with 6E10 antibody (brown) and anti‐lysozyme (green). The white box shows one single amyloid plaque in magnification. Lysozyme is present inside the plaque (white arrow). Green circular structures (black arrow) are cells stained with intracellular lysozyme.

Next, lysozyme protein levels in WT and transgenic mice, harbouring PS1 and the AβPP_Swe_ mutations, were investigated using western blot. AβPP‐mutated mice had an increased protein expression of Aβ and of lysozyme as compared to WT mice, which is consistent with the mRNA expression analysis of lysozyme (Fig. 2A,B). To study whether lysozyme and Aβ colocalized in plaques, immunohistochemistry was performed on frozen tissue from mice with the AβPP_Swe_ mutation. Double‐labelling with antibodies against lysozyme and Aβ showed that lysozyme localized within the plaque as scattered dots (Fig. 2C).

### Lysozyme is increased in the human AD brain

**Figure 3:**
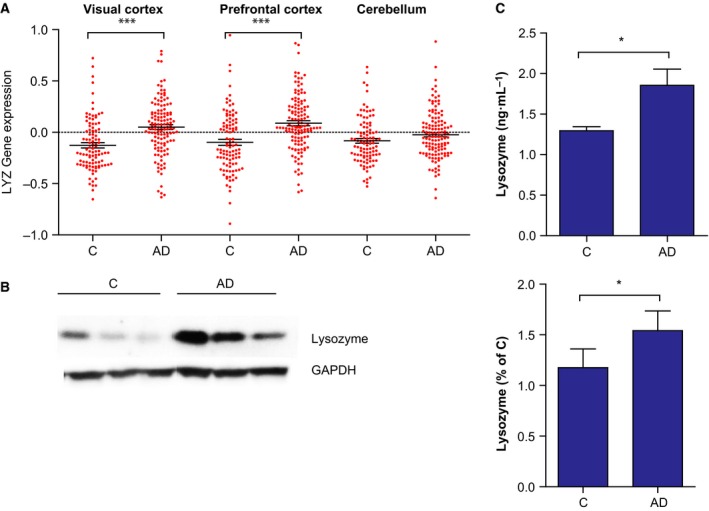
Lysozyme is increased in brains from AD patients. (A) Lysozyme mRNA expression (LYZ; log‐values of the mean intensities, normalized to the average intensities of all samples) in the visual cortex, prefrontal cortex and cerebellum of healthy controls (C) (*n* = 173) and AD patients (*n* = 376). Significant differences were determined by Student's *t*‐test; ***P ≤ 0.001. Bars represent mean ± SEM. (B) Representative western blot of human temporal cortex tissue from healthy controls (Braak 0‐IV,* n* = 24) and AD patients (Braak V‐VI,* n* = 10). Shown are densitometric quantifications of the western blots, normalized to GAPDH levels and to a standard sample loaded on each gel. Significant differences were determined by the nonparametric Mann–Whitney *U* test. Bars represent the mean ± SD. (C) Lysozyme concentrations in CSF from controls (*n* = 25) and biochemical and clinical diagnosed AD patients (*n* = 25) measured using the Meso Scale Discovery technique. Significant differences was determined by Student's *t*‐test, *P ≤ 0.05. Bars represent the mean ± SD.

To study if the increased lysozyme mRNA expression levels in AD mice also applied to human AD patients, a database with data from autopsied tissues from visual cortex, dorsolateral prefrontal cortex and cerebellum was used 16. The mRNA expression of lysozyme was significantly increased in visual cortex and prefrontal cortex of AD patients compared to control, but no difference was seen in cerebellum (Fig. 3A). To study if the protein expression levels of lysozyme were affected in AD, human post‐mortem tissue from temporal cortex was used. The level of lysozyme was significantly increased in the AD group (Braak stages V‐VI) compared to controls (Braak stages 0‐IV) (Fig. 3B), which corresponds with the lysozyme mRNA expression analysis. In addition, lysozyme levels were investigated in an AD validated cohort of CSF and the lysozyme levels were significantly higher in CSF samples from AD patients compared to controls (Fig. 3C).

### Lysozyme protects from Aβ‐induced toxicity in transgenic AD flies

**Figure 4:**
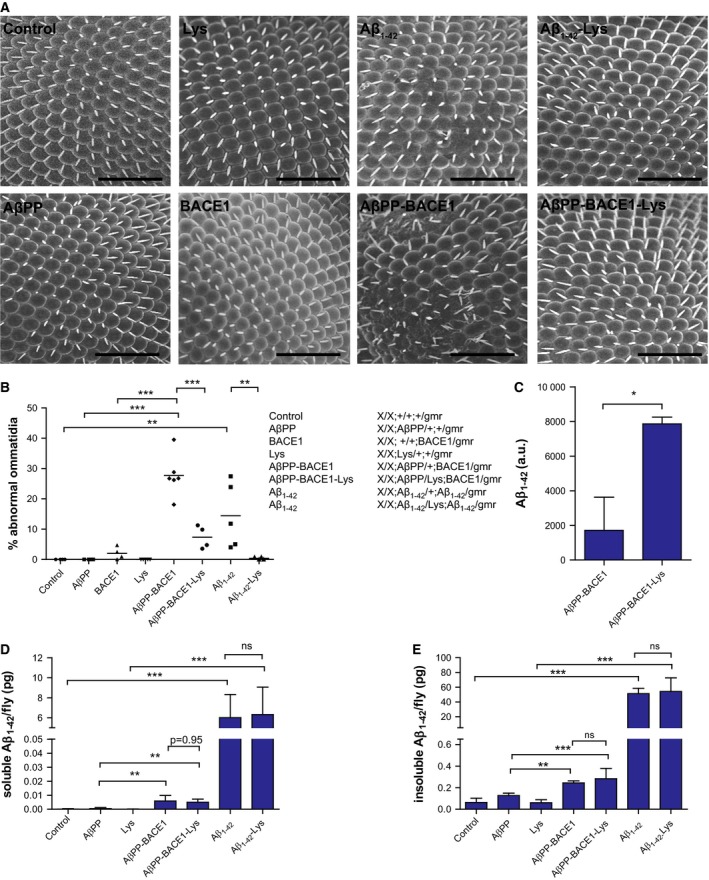
Lysozyme protects transgenic AD flies from Aβ‐induced toxicity. (A) Images of rough eye phenotype obtained by scanning electron microscopy of two AD *Drosophila* models expressing Aβ_1‐42_ or AβPP–BACE1 with or without lysozyme (Lys) and controls; only expressing Gal4, AβPP and Lys at the day of eclosion (150× magnification). Scale bars = 50 μm. (B) Quantification of the abnormal ommatidia seen in A (*n* ≥ 4 of each genotype). All samples are related to their respective control. Significant differences were determined by one‐way ANOVA followed by Tukey's test, **P ≤ 0.01, ***P ≤ 0.001. Meso Scale Discovery analyses performed on fly heads from flies at the day of eclosion to quantify the levels of (D) soluble Aβ_1‐42_ (*n* = 4) and (E) insoluble Aβ_1‐42_ (*n* = 4). All samples are compared to their respective control. Significant differences were determined by one‐way ANOVA followed by Tukey's test, **P ≤ 0.01, ***P ≤ 0.001. (C) Immunoprecipitation assay of extract from AβPP–BACE1 flies with and without coexpression of lysozyme. Brain homogenates were immunoprecipitated with lysozyme antibodies bound to resin and the urea‐eluted samples were analysed for Aβ_1‐42_ using the Meso Scale Discovery technique. The assay was performed on 100 flies of each genotype. Significant differences were determined by Student's *t*‐test, *P ≤ 0.05.

In order to study if lysozyme has any effect on Aβ toxicity, we used one *Drosophila* model where human lysozyme was coexpressed with Aβ_1‐42_ and one, recently characterized, novel *Drosophila* model that coexpressed AβPP and BACE1 (AβPP–BACE1) 17 with or without lysozyme in the eyes of the flies using the retina‐specific *gmr*‐Gal4 driver. The eye morphology was examined at the day of eclosion using SEM. Aβ_1‐42_ flies had a disturbed pattern of ommatidia compared to control flies (only expressing Gal4) and lysozyme flies (Fig. 4A,B). This disturbed ommatidia phenotype was completely rescued when Aβ_1‐42_ was coexpressed with lysozyme (Fig. 4A,B). AβPP–BACE1 flies showed a heavily disturbed pattern of ommatidia with fused and irregular‐shaped ommatidia (Fig. 4A,B), while flies that expressed AβPP demonstrated a regular and symmetric pattern of ommatidia and BACE1 flies exhibited a small disruption of the eye phenotype (Fig. 4A,B). Coexpression of lysozyme with the AβPP–BACE1 flies significantly rescued the disturbed ommatidia phenotype (Fig. 4A,B).

### Lysozyme does not change the level of Aβ but binds to Aβ in the *Drosophila* eye

Having established that lysozyme has a protective effect in the AD transgenic flies, we next investigated if coexpression of lysozyme in these flies could change the level of soluble and insoluble Aβ_1‐42_. The level of Aβ_1‐42_ in the heads of the flies was measured by the Meso Scale Discovery technique. There was a significant increase in both soluble and insoluble levels of Aβ_1‐42_ in flies that expressed Aβ_1‐42_ or AβPP and BACE1 compared to their controls (only expressing Gal4 or AβPP respectively) (Fig. 4D,E). The proportion of insoluble and soluble Aβ_1‐42_ were compared in the flies and the level of insoluble Aβ_1‐42_ was eight times higher than soluble Aβ_1‐42_ in the Aβ_1‐42_ flies and the level of insoluble Aβ_1‐42_ was 40 times higher than soluble Aβ_1‐42_ in the AβPP–BACE1‐expressing flies. Coexpression of lysozyme in the flies that expressed Aβ_1‐42_ or AβPP–BACE1 did not change the soluble or insoluble level of Aβ_1‐42_ in the two fly models (Fig. 4D,E). In order to investigate a potential interaction between Aβ_1‐42_ and lysozyme, an immunoprecipitation assay was performed. Extract from AβPP–BACE1 flies with and without coexpression of lysozyme was run on a column with lysozyme antibodies bound to the resin. The urea‐eluted samples were analysed for Aβ_1‐42_ using the Meso Scale Discovery technique. The AβPP–BACE1–lysozyme extract had a significantly higher Aβ_1‐42_ signal compared to the Aβ_1‐42_ signal in the AβPP–BACE1 extract (Fig. 4C), which indicates an interaction between Aβ_1‐42_ and lysozyme in the AβPP–BACE1–lysozyme flies. Taken together, these results demonstrate that the protective effect of lysozyme overexpression in the AD transgenic flies was not due to changed levels of Aβ_1‐42_, but might instead depend on interactions of Aβ_1‐42_ and lysozyme in the AD fly eye.

### Lysozyme rescues Aβ_Arc_ flies without changing the level of Aβ_Arc_

**Figure 5:**
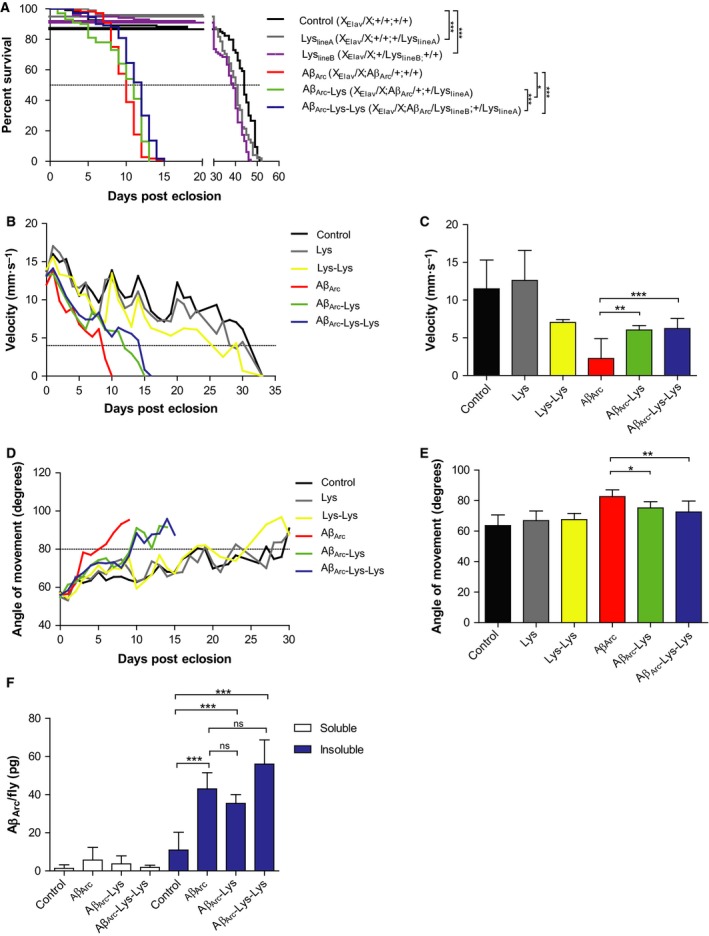
Longevity and behavioural analyses show beneficial effects of lysozyme on Aβ_Arc_ flies. (A) Lifespan trajectories for *Drosophila* flies expressing Aβ with the Arctic mutation (Aβ_Arc_) in the CNS, in the absence or presence of lysozyme expressed as one copy or as two copies compared to control flies (*n* = 100). Kaplan–Meier graph shows per cent survival against age of flies in days after eclosion. Significant differences were determined by log‐rank analysis, *P ≤ 0.05 and ***P ≤ 0.001. The fly behaviour was analysed for control flies and Aβ_Arc_ flies with or without coexpression of one or two copies of lysozyme by (B) velocity measurement, (C) velocity measurement at day 9, (D) angle of movement analysis and (E) angle of movement analysis at day 6. Significant differences were determined by one‐way ANOVA followed by Tukey's test, *P ≤ 0.05, **P ≤ 0.01, ***P ≤ 0.001. Error bars represent mean ± SD,* n* = 30. (F) Meso Scale Discovery analysis performed on the fly heads of control, Aβ_Arc_, Aβ_Arc_‐Lys and Aβ_Arc_‐Lys‐Lys at day 10 after eclosion to quantify the levels of soluble and insoluble Aβ_Arc_. Significant differences were determined by one‐way ANOVA followed by Tukey's test, ***P ≤ 0.001. Error bars represent mean ± SD,* n* = 4.

To further investigate the beneficial effects of lysozyme on Aβ‐induced toxicity, a longevity assay was performed using a *Drosophila* model expressing Aβ_1‐42_ with the Arctic mutation (Aβ_Arc_) with or without coexpressing human lysozyme in the fly CNS using the *elav*‐Gal4 driver. As seen in Fig. 5A, there was a dose‐dependent rescue mediated by lysozyme where the median survival for Aβ_Arc_ flies was prolonged from 10 to 11 days for Aβ_Arc_ flies carrying one copy of lysozyme and from 10 to 12 days for Aβ_Arc_ flies carrying two copies of lysozyme. Flies expressing both lysozyme lines reduced the median survival for the control Gal4 expressing flies from 44 days to 41 and 39 days, line A and line B respectively. Next, the velocity and angle of movement were examined using the iFly technique 18. To study the effect of lysozyme on toxicity induced by the Aβ_Arc_ peptide in adult flies, crosses were set up using the lower temperature 18 °C, which has been demonstrated to significantly lower protein expression 19. The flies were then moved to 29 °C after eclosion to induce expression of Aβ_Arc_ as well as lysozyme. The velocity of the flies will decrease as they age or get sick. Shortly before the flies die, they become immobile and their velocity cannot be recorded; thus, a cut‐off value of 4 mm·s^−1^ was set as an indication of dysfunctional locomotor behaviour 10. The Aβ_Arc_ flies reached below this value after 9 days and after 10 days, no movement could be recorded. For Aβ_Arc_ flies coexpressing one or two copies of lysozyme, the cut‐off value was reached after 12 and 15 days respectively, revealing a dose‐dependent rescue effect of lysozyme on Aβ_Arc_ toxicity where the dysfunctional locomotor behaviour of the Aβ_Arc_ flies was postponed 3 and 6 days (Fig. 5B). For control flies only expressing Gal4 and for flies expressing one or two copies of lysozyme, the cut‐off value was reached after 33, 27 and 28 days, respectively, revealing a detrimental effect of lysozyme on the locomotor performance of the Gal4‐expressing flies (Fig. 5B). The Aβ_Arc_ flies coexpressing one or two copies of lysozyme had a significantly higher velocity compared with the Aβ_Arc_ flies at day 9 (Fig. 5C).

The angle of movement increases as the flies age or get sick and a cut‐off value of 80° was set in order to indicate dysfunction of the flies’ locomotor behaviour 10. In the angle of movement analysis, Aβ_Arc_ flies reached above 80° after 6 days, while Aβ_Arc_ flies with one or two copies of lysozyme both reached above this value after 10 days (Fig. 5D). Control Gal 4 flies and flies coexpressing one or two copies of lysozyme did not display this dysfunction, all genotypes reached above the cut‐off value after 18 days. The Aβ_Arc_ flies coexpressing one or two copies of lysozyme had a significantly lower angle of movement compared with the Aβ_Arc_ flies at day 6 (Fig. 5E).

In order to investigate whether coexpression of one or two copies of lysozyme in the Aβ_Arc_ flies had any effect on the levels of soluble and insoluble Aβ_Arc_, the level of Aβ_Arc_ was measured in the head of the flies using the Meso Scale Discovery technique after ageing the flies for 10 days at 29 °C after eclosion. The soluble levels of Aβ_Arc_ were low and no significant differences were detected between the different fly variants (Fig. 5F). When analysing the insoluble levels of Aβ_Arc,_ significant levels were detected in all Aβ_Arc_ expressing flies compared to control Gal4 flies coexpressing two copies of lysozyme (Fig. 5F). No significant differences were detected between Aβ_Arc_ flies and flies coexpressing the Aβ_Arc_ peptide with one or two copies of lysozyme (Fig. 5F). Taken together, these data show that lysozyme had a dose‐dependent rescue effect on the Aβ_Arc_ toxicity when lysozyme expression was directed to the CNS, lysozyme increased the median survival and decreased dysfunctional locomotor behaviour observed for the Aβ_Arc_ flies, without changing the levels of soluble or insoluble Aβ_Arc_.

## Discussion

Previously, it was demonstrated that lysozyme is associated with Aβ‐plaques in human brain tissue 10. This study demonstrates that both mRNA and protein levels of lysozyme were upregulated in AD mouse models and in human AD cases and that lysozyme associated with Aβ‐plaques in mice brain tissue. In the AD transgenic mouse models, hippocampus and cortex, but not cerebellum, displayed increased mRNA expression of lysozyme compared to WT mice. A similar lysozyme mRNA profile was detected in humans, with increased lysozyme expression in visual cortex and prefrontal cortex in AD patients, and again, no apparent change in cerebellum. The spread of Aβ pathology starts in the areas of cortex and hippocampus and does not affect the cerebellum until late in the disease in both AD mouse models and AD patients 20, 21, 22. In this study, a similar pattern of lysozyme expression was revealed both in the AD mouse models and AD patients. The expression of lysozyme in the cortex and hippocampus of AD transgenic mice correlated well with Aβ pathology and the Aβ pathology had a spatial similarity with the increase of lysozyme expression. It was recently shown that exposure of neuroglia cells to oligomeric Aβ causes an intracellular upregulation of lysozyme as well as an increased secretion of lysozyme 10. Increased lysozyme protein levels were found in the cortex of PS1 and AβPP_Swe_ transgenic mice and in the temporal cortex of human AD patients. An increased level of lysozyme was also detected in CSF from AD patients. This was previously seen in two cohorts of CSF of biochemical diagnosed AD patients 10. The CSF cohort used in this study was from both clinical and biochemical diagnosed AD patients. It is likely that the increased mRNA expression in affected brain regions gave rise to increased protein expression and was responsible for the increased protein level of lysozyme seen in CSF from AD patients. Thus, these *in vivo* and cellular results suggest that Aβ triggers an increase of lysozyme expression and secretion. The expression of lysozyme in tau transgenic mice did not correlate with the age‐related increase in tangle load, as demonstrated by the sigmoidal relationship between levels of Aβ and lysozyme. Tau pathology was increased at 8 months in the tau transgenic mice, both in cortex and hippocampus, but the increase in lysozyme did not appear until after 18 months. These results agree with the previous findings in the AD transgenic mouse models that amyloid plaque pathology correlates with overexpression of several immune genes, whereas this correlation was not found for the tau transgenic mouse model 15.

It was previously shown that lysozyme is able to rescue a *Drosophila* AD model where Aβ_1‐42_ and lysozyme were expressed and secreted, using a secretion tag, in the CNS of the flies 10. In this study, the Aβ_1‐42_ production was targeted to the retina of the flies by use of the *gmr*‐Gal4 driver. The benefit of this driver is that the eye is a nonessential organ for fly survival, and products from transgenes that interfere with ommatidia development can easily be visualized in the fly eye as a rough eye phenotype. Moreover, in this study, we further investigated the effect of lysozyme on Aβ toxicity by introducing a new AD model where the Aβ peptide was generated by the processing of AβPP with β‐secretase followed by γ‐secretase. This new AD fly model is more physiological relevant and produces Aβ of various lengths 17. Flies that expressed Aβ_1‐42_ and flies that coexpressed AβPP and BACE1 had defects in the ommatidia structure. This phenotype was completely abolished when lysozyme was coexpressed, resulting in fly eyes with well‐ordered ommatidia similar to control flies. Thus, in both AD models, the eye phenotype was rescued by coexpression of lysozyme which demonstrates that lysozyme was able to exert its antitoxic effect, both when the Aβ peptide was expressed directly from the transgene and when it was generated by AβPP processing. In addition, the rescue effect of lysozyme on the AβPP–BACE flies reveals possible interactions of lysozyme with several Aβ species generated in these flies.

In addition, we report that lysozyme also had a dose‐dependent protective effect on the Arctic mutation of Aβ_1‐42_, when expressed in the CNS of the flies. The Arctic mutation of Aβ_1‐42_ leads to an increased propensity of Aβ to form oligomeric species and at a faster rate 23. One copy of lysozyme increased the median survival of flies expressing Aβ_Arc_ with 1 day, and two copies extended the median survival with 2 days. To achieve a more complete picture of the health of the flies, a locomotor assay was used to study the velocity and the angle of movement of flies expressing the Aβ_Arc_ peptide alone or together with one or two copies of lysozyme. Coexpression of both one and two copies of lysozyme demonstrated beneficial effects on both the velocity and angle of movement of the Aβ_Arc_ flies, which proves that lysozyme has the ability to decrease locomotor disability caused by the Aβ_Arc_ peptide.

The rescue effect of lysozyme on Aβ_Arc_ toxicity in the longevity and locomotor assays cannot be attributed to a general beneficial effect of the fly health by expressing lysozyme in the fly CNS as flies only expressing lysozyme showed a reduction in the median survival time compared to Gal4 control flies (by approximately 4 days). A detrimental effect was also detected in the velocity experiment for lysozyme expressing flies compared to the Gal4 flies, while no general effect of lysozyme was detected in the angle of movement analysis of Gal4 flies. Altogether, these results demonstrate that lysozyme has a protective effect on Aβ‐induced toxicity.

One possible explanation for the protective action of lysozyme could be that it increases the degradation of the Aβ peptide in the flies. This was shown in a study where coexpression of lysozyme in Aβ_1‐42_ transgenic *Drosophila* increased the survival of the flies and reduced the levels of Aβ_1‐42_ 10. However, in this study, there was no difference in the amount of either soluble or insoluble Aβ_1‐42_ between flies that expressed Aβ_1‐42_ or Aβ_1‐42_ coexpressed with lysozyme, or AβPP–BACE1 flies and flies with AβPP–BACE1 and lysozyme. The discrepancies between these studies are that in the previous study, Aβ_1‐42_ and lysozyme were expressed in the CNS but in this study, the expression was targeted to the eyes of the flies. The degradative capacity of Aβ_1‐42_ in the brain and eye can be different and lysozyme can interact with Aβ_1‐42_ in another fashion in the brain compared with the eye, which might explain the discrepancies between the results.

We have previously shown *in vitro* that lysozyme binds to monomeric Aβ_1‐42_ and prevents the formation of toxic Aβ_1‐42_ species by reducing the aggregation propensity of Aβ_1‐42_ 10. Other studies have shown that lysozyme also inhibits the aggregation of Aβ_1‐40_ and Aβ_17‐42_ by binding during the early stages of fibrillation, thereby reducing toxic species and cytotoxicity 11, 12, 24. We have also reported that lysozyme colocalizes with Aβ plaques in human AD brain and with Aβ aggregates in Aβ‐overexpressing *Drosophila* flies 10. In this study, we show that lysozyme stains plaques in AβPP mice which indicate that fibrillar Aβ and lysozyme are in close association. To investigate if lysozyme was able to bind Aβ in the *gmr*‐Gal4‐derived flies, immunoprecipitation capturing lysozyme was performed on fly head homogenates followed by Meso Scale Discovery, where Aβ was detected with the 6E10 antibody. Indeed, there was a significant increase of Aβ_1‐42_ in the homogenates from AβPP–BACE1–lysozyme flies compared with the AβPP–BACE1 flies. This demonstrates that also in this *Drosophila* model lysozyme was able to interact with Aβ and thus likely rescues the degeneration of the ommatidia in the fly eye by preventing the formation of cytotoxic Aβ species.

When quantifying the soluble and insoluble levels of Aβ_Arc_ in the fly CNS, only insoluble Aβ species were detected and no significant differences could be observed between the insoluble levels in flies, solely expressing Aβ_Arc_, and in flies where Aβ_Arc_ was coexpressed with one or two copies of lysozyme. Hence, the protective properties of lysozyme in the longevity and locomotor analyses of the Aβ_Arc_ flies must be unrelated to degradation of Aβ_Arc_. A possible explanation for the antitoxic effect of lysozyme in the Aβ_Arc_ flies could be due to interactions between the insoluble Aβ_Arc_ species and lysozyme reducing the toxicity of these species. The Arctic mutation causes the Aβ_1‐42_ peptide to be more aggregation prone and therefore less prone to be degraded. This might explain why lysozyme overexpression in the CNS of Aβ_Arc_ flies was unable to cause an increased degradation of Aβ, which earlier was suggested for lysozyme overexpression in the CNS of Aβ_1‐42_ flies 10.

In conclusion, we show that the expression of lysozyme was increased in the brains of both transgenic AD mice and humans with AD. Furthermore, we demonstrate favourable effects of lysozyme when expressed in different *Drosophila* models of AD. In flies that expressed Aβ_1‐42_ or AβPP together with BACE1 in the eyes, the rough eye phenotype indicative of toxicity was completely rescued by coexpression of lysozyme. In *Drosophila* flies that express the toxic Aβ_Arc_, lysozyme both increased the fly survival dose dependently and decreased dysfunction in locomotor behaviour. We propose that this is due to binding of lysozyme to toxic species of Aβ which prevent these from exerting their toxic effect. Thus, the upregulated of lysozyme expression in AD patients and transgenic AD mice might be a rescue response towards Aβ toxicity. These results highlight the possibility of lysozyme as a potential therapeutic target for AD.

## Materials and methods

### Gene expression studies

The database (www.mouseac.org) which includes microarray data from amyloid transgenic mice carrying the AβPP_Swe_ mutation and/or PSEN1 (PSEN1: M146V) either heterozygous or homozygous, and tau transgenic mice (TAU: P301L) 15, was used to study lysozyme mRNA expression and pathology during ageing (GEO accession number GSE64398). In addition, a microarray data set containing information about human mRNA expression was used to study the lysozyme expression in 101 nondemented healthy controls and 129 AD patients (GEO accession number GSE44772) 16.

### Protein extraction from brain tissue

Human brain tissue were obtained from the Sydney Brain Bank at Neuroscience Research Australia and the New South Wales Tissue Resource Centre at the University of Sydney and characterized according to established neuropathological criteria 25. Informed consent for the collection of material was obtained prior to death and tissue use was approved by the University of New South Wales Human Research Ethics Committee. Brain biopsies from 34 cases (Braak stages 0‐VI) from temporal cortex were frozen with a post‐mortem delay of maximum 64 h. The tissues were homogenized in 4 μL·mg ^−1^ sample of cold TBS [50 mm TRIS, 125 mm NaCl, 5 mm EDTA, protease inhibitor (Roche, Basel, Switzerland)] using a motorized pestle prior to centrifugation at 20 800 ***g*** at 4 °C for 1.5 h.

The APP‐PS1 AD mouse model expressing chimeric mouse/human APP695swe/Swedish mutations (K595N/M596L) and mutant human PS1 (PS1/∆E9) was obtained from the Jackson Laboratory (Bar Harbor, ME, USA; Strain name, B6.Cg‐Tg AβPPswePSEN1dE9) 85Dbo/J; Stock #005864). Animal ethics approval was from the University of Wollongong Animal Ethics Committee (AE11/03). The mice were aged for 12 months without any intervention; food and water were available *ad libitum*. In brief, 12‐month‐old APP‐PS1 and age‐matched wild‐type mice were euthanized by CO_2_ asphyxiation to ensure that the mice did not suffer unnecessarily, and transcardially perfused with ice‐cold phosphate‐buffered saline (PBS). The right hemisphere from the APP‐PS1 and age‐matched wild‐type mice were snap frozen and stored at −80 °C. Cortex was dissected and homogenized in 10 volumes of 140 mm NaCl, 3 mm KCl, 25 mm Tris (pH 7.4), containing 1% Nonidet P‐40 and Roche complete protease inhibitors using a Precellys 24 homogenizer (2 × 30 s, 6000 g). The supernatants were saved and the protein concentration was determined using a DC protein assay (Bio‐Rad, Hempstead, UK).

### CSF cohort

Deidentified and archived CSF samples, that were both biochemically and clinically diagnosed with AD (*n* = 25), were age‐ and gender‐matched with controls with normal levels of the CSF AD biomarkers P‐tau_181P_, T‐tau and Aβ_1‐42_ (*n* = 25). The samples were provided by the Clinical Neurochemistry Laboratory, Sahlgrenska University Hospital/Mölndal, Sweden. The study was approved by the Ethical committee at the University of Gothenburg. For more information regarding CSF handling and P‐tau_181P_, T‐tau and Aβ_1‐42_ analysis, see previously described methods 26.

### Western blotting

Protein separation was performed as described previously 26. Forty microgram of total protein was mixed with loading buffer and blotted onto a nitrocellulose membrane using an iBlot Dry Blotting System (Invitrogen, Paisley, UK) and incubated with polyclonal rabbit anti‐human lysozyme antibody (1 : 2000) (Dako, Glostrup, Denmark) overnight in 4 °C, followed by horseradish peroxidase (HRP)‐linked goat anti‐rabbit antibody (Dako) for 1 h at room temperature. Mouse anti‐glyceraldehyde‐3‐phosphate dehydrogenase (GAPDH; 1 : 20 000; Novus Biologicals, Littletown, CO, USA) was used as loading control. To correct for differing protein levels between blots, all samples were normalized to a reference sample, which was loaded onto each gel.

### Immunohistochemistry

C57Bl/6 transgenic AβPP_Swe_ mice (kindly provided by Professor Lars Nilsson at Oslo University) were housed in a pathogen‐free environment on a 12‐h light/dark cycle. The mice were aged for 15 months without any intervention; food and water were available *ad libitum*. The experimental procedures were performed in accordance with the Animal Care and Use Ethical Committee at the Linköping University (ethical registration number 84‐12) which follow the directives 2010/63/EU of the European Parliament and of the Council of 22 September 2010 on the protection of animals used for scientific purposes. The mice were euthanized by CO_2_ asphyxiation to ensure that the mice did not suffer unnecessarily, and transcardially perfused with saline solution (NaCl, 0.9%) before dissection of the brain. The brain tissue were stored in formalin, dehydrated in sucrose, sectioned and frozen. Immunohistochemistry was performed according to the manufacturer's protocol (Biocare Medical, Concord, CA, USA). Aβ plaques were immunostained using the 6E10 antibody (1 : 100; Signet Laboratories/Covance, Dedham, MA, USA), then colour was developed using diaminobenzidine (DAB; Biocare Medical) and lysozyme was immunostained with an anti‐lysozyme antibody (1 : 100; Dako) and colour was developed with Vina Green^™^ Chromogen Kit (Biocare Medical). To stop cross‐reaction, the brain slices were incubated with denaturing solution (Biocare Medical) for 5 min before double staining with lysozyme antibody.

### *Drosophila* stocks

To direct tissue‐specific protein expression in UAS transgenic *Drosophila melanogaster,* the Gal4/UAS system was used 27. In the SEM analysis, the tissue‐specific *gmr*‐Gal4 driver strain was used to direct protein expression to the photoreceptors present in the retina of the flies 28. Control *w* ^1118^ flies (only expressing Gal4), AβPP_695_ (AβPP) and BACE1‐expressing flies were purchased from Bloomington stock centre. Signal‐peptide‐Aβ_1‐42_ flies and signal‐peptide‐Aβ_Arc_ (E22G) were kindly provided by Professor Damian Crowther 29. Lysozyme‐expressing flies were constructed as described previously 30. To generate flies coexpressing Aβ_1‐42_ or AβPP–BACE1 together with lysozyme or flies coexpressing Aβ_Arc_ with either one or two copies of WT lysozyme, cross‐breeds were set up in multiple steps with 12:12 h light:dark cycles and 60% humidity. In the longevity and locomotor analyses, the *elav*‐Gal4 driver line was used to direct protein expression to the fly CNS. To generate X_Elav_/Y;Aβ_Arc_/Ife;TM6b/+, a cross was set up using X_Elav_/X_Elav_;CyO/Ife;TM6b/mkrs and X/Y;Aβ_Arc_/Aβ_Arc_;+/+. In order to achieve coexpression of Aβ_Arc_ with one or two copies of lysozyme, males generated in the cross previously described were crossed with X/X;Lys_lineB_/Lys_lineB_;+/+ or X/X;Lys_lineB_/Lys_lineB_;Lys_lineA_/TM6b. All gene constructs of lysozyme and Aβ_Arc_ where cloned into the Gal4‐responsive pUAST expression vector to generate UAS‐lysozyme or UAS‐arctic transgene. These constructs were injected into the same background *W* ^1118^ flies 29, 30.

### SEM of eye phenotype

Cross‐breeds were set up at 25 °C. At the day of eclosion, flies were euthanized with ether before they were mounted onto 12‐mm aluminium specimen stubs with an adhesive tape and air‐dried for 24 h. The samples were sputter coated with 15 nm of platinum and stored in a vacuum desiccator prior to analysis using SEM (JEOL JSM‐6320F). The eyes were scanned at 150× magnification at an accelerating voltage of 10 kV. Images were recorded using the SemA for 5.21 digitizer system (Insinooritoimisto Rimppi Oy). In a blinded set‐up, the images were printed and assigned a square of approximately 100 ommatidia in the centre of the eye. All abnormal ommatidia were quantified within this square and further related to all ommatidia.

### Sample preparation for Aβ_1‐42_ quantification

Cross‐breeds were set up at 25 °C. Flies were snap frozen in liquid nitrogen at the day of eclosion. Approximately, 20 heads per genotype were homogenized in 120‐μL soluble extraction buffer [50 mm Hepes, 5 mm EDTA, protease inhibitor (Complete EDTA‐free Protease Inhibitor Cocktail Tablets, Roche Diagnostic)] for the soluble fraction of Aβ_1‐42_. After homogenization, the samples were centrifuged for 5 min, 16 000 ***g***. The supernatant was then collected in an Eppendorf tube. The pellet was rehomogenized in 25‐μL insoluble extraction buffer (50 mm Hepes, 5 mm EDTA, 5 m guanidinium chloride, protease inhibitor) for the insoluble fraction of Aβ_1‐42_. After homogenization, the samples were incubated at RT for 10 min, followed by sonication in a water bath for 4 min. The samples were then centrifuged for 10 min, the supernatant of the insoluble fraction was collected and diluted 1 : 10 in Diluent 35 (R50AE‐2, Meso Scale Discovery, Rockville, ML, USA). The samples were stored at −80 °C. To account for differences in the protein extraction step, the total amount of protein extracted was quantified using the Bio‐Rad DC Protein Assay Kit II (500‐0112, Bio‐Rad).

### Quantification of Aβ_1‐42_ by Meso Scale Discovery analysis

The wells in a multi‐spot 96‐well V‐PLEX human Aβ_1‐42_ (K151LBE‐1, Meso Scale Discovery) were blocked using 150‐μL Diluent 35 for 1 h at RT with gentle agitation. After blocking, 50 μL of each prepared protein sample was added to the wells in duplicates (incubated 1 h, RT, gentle agitation). After sample incubation, the wells were washed in 3 × 150 μL PBS‐T before adding 25 μL of the detection antibody (50× Sulfo tag 6E10, Meso Scale Discovery) (1 h, RT, gentle agitation). The wells were then washed again (3 × 150 μL PBS‐T) before adding 150 μL 2× read buffer (10 min incubation, RT, no agitation). The plate was analysed using a SECTOR Imager 2400 instrument (Meso Scale Discovery).

### Sample preparation and quantification of Aβ_Arc_ by Meso Scale Discovery

Fly crosses were set up at 18 °C and at the day of eclosion moved to 29 °C where they were aged for 10 days. After ageing, 10 fly heads of each genotype were placed in Eppendorf tubes and stored at –80 °C until use. The Meso Scale Discovery analysis was performed as previously described by Caesar *et al*. 31. To account for any differences in the protein extraction step, the total amount of protein extracted for each sample was quantified using the Bio‐Rad DC Protein Assay Kit II (500–0112, Bio‐Rad).

### Longevity assay

Fly crosses using the *elav*‐Gal4 driver were set up at 29 °C and maintained at 29 °C (60% humidity, 12:12 h dark:light cycle) after eclosion. A set of 100 female offspring for each genotype was collected at the day of eclosion. The flies were then divided into groups of approximately 10 flies and placed in vials containing agar food (20 g agar, 20 g sugar·L^−1^ dH_2_O) and yeast paste (dry baker's yeast mixed with water). Every 2–3 day, the flies were transferred to vials containing fresh food and the number of living flies was counted. This was repeated until all flies had died. Kaplan–Meier survival curves were generated for lifespan assessment.

### Locomotor assay

Fly crosses were set up at 18 °C and at the day of eclosion, moved to 29 °C. Sets of 30 female flies of each genotype were collected and divided into groups of 10 and placed in vials containing fly food. To analyse the flies’ locomotor behaviour, the flies were filmed during 90 s and tapped to the bottom of the vial every 30 s to reactivate locomotor behaviour. This was carried out every day until all Aβ_Arc_‐expressing flies had stopped to move; the Gal4‐ and lysozyme‐expressing flies were analysed every other/third day until no locomotor behaviour could be detected for these fly variants. The videos were processed and analysed using the ifly software 18, which calculated the velocities and angles of movement generated by the flies in each recorded video clip. The locomotor measurements were carried out as described previously 17.

### Immunoprecipitation

Lysozyme‐specific capture resin was prepared as described earlier 30 with CNBr‐activated Sepharose 4B resin (GE Healthcare, Pittsburgh, PA, USA) and 0.5 mg·mL^−1^ polyclonal rabbit anti‐human lysozyme (Dako; A0099). One hundred microlitre of the anti‐lysozyme conjugated medium slurry was placed in a Micro Bio‐Spin Chromatography Column (Bio‐Rad) and the column was equilibrated with SuperBlock TBS Blocking Buffer (Thermo Scientific Pierce, Rockville, IL, USA) supplemented with a protease inhibitor (TBS‐PI). Heads from AβPP flies, AβPP–BACE flies and AβPP–BACE flies with lysozyme were homogenized with a pestle in 200 μL TBS‐PI and centrifuged for 1 min at 16 000 ***g***. One hundred flies of each genotype were used. The supernatant was centrifuged a second time before incubation in the column for 5 min and centrifuged 160 ***g*** for 1 min. The resin was then washed four times with TBS‐PI before lysozyme was eluted with 6 m urea. The concentration of Aβ_1‐42_ was measured using the Meso Scale Discovery technique, as described above.

### Statistical analysis

All statistical analyses and graphs were performed and drawn using graphpad prism software v. 7 (GraphPad Software, La Jolla, CA, USA). Two‐way ANOVA followed by Tukey's *post hoc* test was used to test for significant differences between multiple groups of mice. Correlation analysis was performed using the Pearson correlation coefficient. One‐way ANOVA followed by Tukey's test was used for quantification of abnormal ommatidia and to test for differences in protein levels analysed with the Meso Scale Discovery technique. Student's *t*‐test or Mann–Whitney *U* test was used to test for significant differences between two groups. For the lifespan assay, Kaplan–Meier survival curves were generated and log‐rank statistical analysis (Mantel–Cox) was performed. Statistical significance was defined for *P*‐values of < 0.05 (*), < 0.01 (**) and < 0.001 (***).

## Author contributions

LS, LB, SN, ACB, KK planned experiments; LS, LB, SN, CK, CJ, LH, HL performed experiments; LS, LB, CK, ACB, KK analysed data; KB, HZ, BG contributed human material; CN contributed mice material; LS, LB, ACB, KK wrote the paper; all authors revised the manuscript critically and approved the final version to be submitted.
